# Modelling COVID-19 severity in the Republic of Ireland using patient co-morbidities, socioeconomic profile and geographic location, February to November 2020

**DOI:** 10.1038/s41598-021-98008-6

**Published:** 2021-09-16

**Authors:** M. Boudou, C. ÓhAiseadha, P. Garvey, J. O’Dwyer, P. Hynds

**Affiliations:** 1 Spatiotemporal Environmental Epidemiology Research (STEER) Group, Environmental Sustainability and Health Institute, Technological University Dublin, Dublin, Ireland; 2 Department of Public Health, Health Service Executive, (HSE), Dublin, Ireland; 3 Health Protection Surveillance Centre (HPSC), Dublin, Ireland; 4 Environmental Research Institute, University College Cork, Cork, Ireland; 5 Irish Centre for Research in Applied Geoscience, University College Dublin, Dublin, Ireland

**Keywords:** Diseases, Health care, Risk factors

## Abstract

Understanding patient progression from symptomatic COVID-19 infection to a severe outcome represents an important tool for improved diagnoses, surveillance, and triage. A series of models have been developed and validated to elucidate hospitalization, admission to an intensive care unit (ICU) and mortality in patients from the Republic of Ireland. This retrospective cohort study of patients with laboratory-confirmed symptomatic COVID-19 infection included data extracted from national COVID-19 surveillance forms (i.e., age, gender, underlying health conditions, occupation) and geographically-referenced potential predictors (i.e., urban/rural classification, socio-economic profile). Generalised linear models and recursive partitioning and regression trees were used to elucidate COVID-19 progression. The incidence of symptomatic infection over the study-period was 0.96% (n = 47,265), of whom 3781 (8%) required hospitalisation, 615 (1.3%) were admitted to ICU and 1326 (2.8%) died. Models demonstrated an increasingly efficacious fit for predicting hospitalization [AUC 0.816 (95% CI 0.809, 0.822)], admission to ICU [AUC 0.885 (95% CI 0.88 0.89)] and death [AUC of 0.955 (95% CI 0.951 0.959)]. Severe obesity (BMI ≥ 40) was identified as a risk factor across all prognostic models; severely obese patients were substantially more likely to receive ICU treatment [OR 19.630] or die [OR 10.802]. Rural living was associated with an increased risk of hospitalization (OR 1.200 (95% CI 1.143–1.261)]. Urban living was associated with ICU admission [OR 1.533 (95% CI 1.606–1.682)]. Models provide approaches for predicting COVID-19 prognoses, allowing for evidence-based decision-making pertaining to targeted non-pharmaceutical interventions, risk-based vaccination priorities and improved patient triage.

## Introduction

Since the first reported national case on February 29th 2020, the Republic of Ireland, alongside much of the world, has endured three waves of COVID-19 infection, and numerous phases of non-pharmaceutical interventions including business, hospitality and school closures, stay at home orders, domestic travel restrictions, and nationwide lockdowns^1,2^. As of early April 2021, approximately 238,000 confirmed infections and 4718 deaths, respectively, have been reported, thus placing unprecedented pressure on critical care services^1^. The clinical manifestations of COVID-19 infection range from asymptomatic infection to pneumonia, which can progress to acute respiratory distress syndrome, multi-organ failure and, ultimately, death^3,4^. Globally, approximately 80% of reported cases are characterised by absent or mild symptoms, while 15–20% progress to severe pneumonia causing death in 1–5% of patients^5,6^.

Monitoring the clinical outcomes of patients diagnosed with COVID-19 is vital to understand the epidemiological and healthcare burden of SARSCoV-2, prioritise high-risk cases in the short term, and perhaps more importantly, provide a robust evidence-base for future public health emergency planning. Several risk factors have been statistically correlated with COVID-19 outcomes within the scientific literature, including age^7^, gender^8^, underlying chronic conditions^9^, race/ethnicity^10^, and occupation^11^. For example, a study cohort of 10,454 COVID-19 patients from Galicia (Spain) reports the presence of seven comorbidities (heart failure, hypertension, rheumatoid arthritis, COPD, asthma, obesity and diabetes) were associated with hospitalisation, three (liver disease, obesity and diabetes) with intensive care unit (ICU) admission, and six (lymphoma/leukaemia, heart disease, dementia, COPD, diabetes and chronic kidney disease) with death^4^. Likewise, a meta-analysis of over 3.1 million reported global cases indicates that male patients exhibit almost three times the odds of requiring ICU admission (OR = 2.84; 95% CI = 2.06, 3.92) and higher odds of death (OR = 1.39; 95% CI = 1.31, 1.47) compared to female patients^8^.

While the abovementioned studies leave little doubt as to the veracity of and necessity for prognostic modelling of COVID-19 outcomes, it is also important to consider the marked variation between regions and their background population health profile (i.e., comorbidity), socioeconomic profile, demographic distribution, and the complex interactions between these potential drivers of severe COVID-19. Accordingly, the current study sought to develop a series of prognostic models to elucidate progression from symptomatic COVID-19 to hospitalization, intensive care and death in the Republic of Ireland. Several case-specific and geographically referenced predictors were employed for model training and testing, including age, gender, comorbidity profile, area-specific socioeconomic components, urban/rural classification and case classification (i.e., sporadic or cluster-associated).

## Methods

### Infection data

Confirmed and anonymised case data were obtained from the Computerised Infectious Disease Reporting (CIDR) database (http://www.hpsc.ie/CIDR/), an information system used for the collation of notifiable (communicable) infection data in Ireland^12^. For the purposes of clarity and comparability, only laboratory-confirmed, symptomatic cases have been included for analyses, that is cases associated with detection of SARS-CoV-2 nucleic acid or antigen in a clinical specimen (laboratory criteria), and exhibiting at least one of the following: sudden onset of cough or fever or shortness of breath or anosmia, ageusia or dysgeusia (clinical criteria) were included for analyses. Primary and secondary case classifications were included as potential predictors, with sporadic (i.e., not recorded as associated with a confirmed outbreak or cluster) and outbreak index cases (the first case identified as part of a recognised outbreak/cluster) were defined as primary cases, while all other known outbreak cases were defined as secondary cases.

All symptomatic COVID-19 cases with an “epi-date” occurring between 29th February and 30th November 2020 were included for analyses. Address level data had already been geocoded to Small Areas by the Health Service Executive (HSE)-Health Intelligence Unit. Research ethical approval for use of the COVID-19 dataset and associated analyses were granted by the National Research Ethics Committee for COVID-19-Related Health Research (NREC COVID-19) (Application number: 20-NREC-COV-061). All research methods including data processing and analyses were performed in accordance with relevant guidelines and regulations. As per conditions of the National Research Ethics Committee for COVID-19-Related Health Research, informed consent from all participants and legal guardians was waived, with data processing and analyses undertaken using irreversibly anonymised data.

### Predictors

#### Comorbidity, underlying health and occupation

All comorbidities included in the “Underlying Clinical Conditions” section of the Health Service Executive (HSE) Health Protection Surveillance Centre (HPSC) COVID-19 Case Form^13^ were extracted for analyses, as follows:

- Chronic heart disease
- Hypertension
- Chronic neurological disease
- Chronic respiratory disease
- Chronic kidney disease
- Chronic liver disease
- Asthma requiring medication
- Immunodeficiency, including HIV
- Diabetes
- Severe obesity (BMI ≥ 40)
- Cancer/Malignancy

The total case-specific comorbidity number was calculated and assigned on a case-wise basis. Data pertaining to an ongoing pregnancy and ≤ 6 weeks post-partum were extracted for all cases. While > 20 occupational classifications were used for reporting, a binary (YN) predictor was created, based on a recent Irish study^11^, to delineate those cases attributed to occupations in healthcare, as this represents a subset associated with particularly high exposure to infection and subsequent serial testing.

#### Urban/rural classification

A categorical Small Area (SA)-specific settlement type variable with three levels of measurement was developed using data obtained from the Irish Central Statistics Office (CSO). The CSO settlement type dataset^14^ comprises six categories classified along an urban/peri-urban/rural scale ranging from ‘city’ (1) to ‘highly rural/remote areas’ (6). The classification variable was coded such that any classification which included a built-up area (classification 1–4) was recoded as ‘urban’, classification 5 (rural areas with high urban influence) was recoded as commuter/peri-urban, with all other areas (classification 6) coded as ‘rural’.

#### Deprivation index and components

The Pobal Haase-Pratschke (HP) Deprivation Index is a composite measure of deprivation/affluence derived from national population census data and comprising 16 individual components, representing three dimensions of deprivation: demographic profile, social class composition, and labour market situation (Table 1)^15^. The absolute deprivation score reflects any changes to the national economy at SA level between census periods while the relative deprivation index score is a comparative measure of deprivation between SAs during a census period^15^. Deprivation indices (absolute and relative) and component scores were obtained for the most recent (2016) national census of Ireland and attributed to all laboratory-confirmed COVID-19 cases.

**Table 1:** Pobal HP deprivation index components and descriptions.

Component Name (Label)	Component Description
Absolute HP Index Score (HPabs)	Composite measure of deprivation calculated for each SA, measured on a single scale across all census periods
Relative HP Index Score (HPrel)	A measure of the level of deprivation in each SA relative to all other small areas surveyed
Total population (TOTPOP)	Total population in each SA during each census period
Population Change (POPCHG)	Percentage increase in population over the previous 5 years
Age dependency rate (AGEDEP)	Percentage of population aged < 15 or > 64 years of age
Lone parent ratio (LONEPA)	Percentage of households with children < 15 years and headed by a single parent
Primary education (EDLOW)	Percentage of people in each SA with primary education as their highest level of education attainment
Third level education (EDHIGH)	Percentage of people in each SA with third level education as their highest level of education attainment
Higher and lower professionals (HLPROF)	Percentage of households headed by professionals or managerial and technical employees, including farmers with ≥ 100 acres
Proportion of semi-skilled/ unskilled workers (LSKILL)	Percentage of households in each SA headed by semi‐skilled or unskilled manual workers, including farmers with < 30 acres
Male unemployment (UNEMPM)	Rate of male unemployment in each SA
Female unemployment (UNEMPF)	Rate of female unemployment in each SA
Persons per room (PEROOM)	Mean number of persons per household room in each small area
Local authority housing (LARENT)	Percentage of local authority housing in each SA
Privately rented housing (PRRENT)	Percentage of privately rented housing in each SA
Own home (OHOUSE)	Percentage of privately owned housing in each SA

### Statistical analysis

To counteract the high proportion of “non-severe” outcomes within the case dataset, a balanced dataset was created via up-sampling. Cases were randomly partitioned into model training (80%) and validation (20%) subsets based on the dependent variable of interest (i.e., Hospital Inpatient, ICU Admission, Mortality), to derive generalised linear models using a binomial link function (i.e., dispersion = 1, parameter number = number of coefficients). Models were trained using all available predictors, with variables individually removed from the model based on the lowest Akaike Information Criterion (AIC) and the least significant variable *p *value (i.e., stepwise approach). Each significant variable was subsequently removed from the model to assess its effect on model accuracy based on developed confusion matrices. Only variables contributing significantly to model accuracy were retained. Receiver operating characteristic (ROC) curves and the area under the curve (AUC) were employed to assess the diagnostic ability of developed models; internal validation was undertaken on calculated AUROCs using 500 bootstrapped samples for model training and validation. The Nagelkerke R^2^ was used to calculate the proportion of explained variance explained by the selected predictors, with the Brier score used to assess model performance (calibration).

The “best predictors” identified via validated GLMs were used to develop “rpart” (Recursive Partitioning and Regression Trees) models to identify individual variable thresholds and the causative pathways from symptomatic infection to each of the three modelled outcomes (i.e., attribute cut-offs (“splitters”) and causative order/importance). As for GLMs, a balanced dataset and partitioning approach (80/20) for training and testing sets were employed. A 10 × cross-validation tree development method was used, with tune length (number of default parameters) varying from 2 to 10 for training. Final models were selected to maximise the complexity/accuracy of the decision trees (based on Cp (complexity parameter)). Accordingly, presented models are those with the maximum number of predictors in concurrence with the highest level of accuracy based on true positives (i.e., sensitivity). Final decision trees are presented to highlight successive thresholds (cut-off values (splitters) for continuous predictors, significant category for categorical predictors, predictor order) and pathways (i.e., predictor order) identified for progression from symptomatic confirmed COVID-19 infection to each of the modelled outcomes.

All statistical analyses were carried out in R version 4.0.3 using the Caret, pROC, deskTOOLS, fmsb, glmnet and randomforest packages. All packages are freely available at http://cran.r-project.org.

## Results

### Descriptive statistics

Overall, 47,265 laboratory-confirmed cases of symptomatic COVID-19 infection (53.4% female; mean age 41.2 years; 0.96% of national population) were included for analyses (Table 2), all of which occurred between February 29th and November 30th 2020. Of these, 3781 (7.99%) were reported as having been hospital inpatients, 615 (1.3%) were admitted to an intensive care unit (ICU) and 1326 (2.8%) died, of whom 599 (45.2%) had not been classified as a hospital inpatient. The odds of progression to severe outcomes typically increased with age, frequency/number of comorbidities, and deprivation elements, for example, across the entire study cohort, 21% of cases (n = 37,341) presented with ≥ 1 underlying clinical condition, compared with 60.4%, 78.9% and 84.2% among hospitalised cases, ICU admissions and deaths, respectively (Table 2). Likewise, mean HP deprivation scores were markedly lower among cases associated with hospitalisation (− 1.82), ICU (− 0.28) and death (− 1.7) than the mean score across all symptomatic cases (0.24). Patients that died in hospital were typically younger (mean 77.3 years vs 84 years), associated with a higher comorbidity score (mean 1.96 vs 1.51) and markedly lower deprivation score (mean − 2.41 vs − 0.84), than those that died outside of hospital.

**Table 2:** Clinical characteristics of symptomatic COVID-19 cases—demographics, comorbidities, mean deprivation scores, and outcomes, February to November 2020.

Parameter	Total	Hospitalised	ICU admission	Mortality
N (%)	47,265	3781 (8)	615 (1.3)	1326 (2.8)
Mean age (years)	41.2	62.2	59.8	80.3
Female (%)	53.4	43.5	31.1	45.7
Chronic heart disease (%)	6.6	29.5	46.5	46.8
Hypertension (%)	6.0	18.2	35.8	19.7
Chronic neurological disease (%)	2.7	10.6	5.0	33.6
Chronic respiratory disease (%)	8.1	19.8	26.5	21.6
Chronic kidney disease (%)	1.5	8.7	9.8	13.7
Chronic liver disease (%)	0.5	2.3	3.4	2.2
Asthma (req medication) (%)	1.1	4.7	12.2	2.9
Immunodeficiency (%)	1.2	3.9	6.8	3.0
Diabetes (%)	3.7	16.0	27.3	17.2
Severe obesity (BMI ≥ 40) (%)	0.6	4.2	18.4	2.8
Cancer/malignancy (%)	2.1	10.8	13.0	17.9
Pregnant (%)	0.8	0.6	0.2	0
≤ 6 weeks post-partum (%)	0.0002	0.1	0.3	0
Healthcare worker (%)	19.6	9.1	9.0	0.5
Urban/commuter/rural (%)	71.8/12.6/15.5	69.9/12.1/18.0	76.6/9.8/13.7	72.9/14.6/12.5
HP deprivation (mean score)	0.24	− 1.82	− 0.28	− 1.7
POPCHG (mean %)	0.07	0.05	0.06	0.08
AGEDEP (mean %)	34.2	35.7	34.4	40.0
LONEPA (mean %)	21.4	22.9	22.0	21.1
EDLOW (mean %)	13.7	15.8	14.0	17.4
EDHIGH (mean %)	36.0	33.0	35.2	33.2
HLPROF (mean %)	34.9	33.2	34.4	34.1
LSKILL (mean %)	18.2	19.5	19.2	18.0
UNEMPM (mean %)	14.6	16.0	15.0	14.2
UNEMPF (mean %)	13.0	13.7	13.5	12.3
PEROOM (mean %)	0.57	0.56	0.56	0.62
LARENT (mean %)	9.5	10.6	11.0	9.3
PRRENT (mean %)	21.9	19.3	21.3	20.0
OHOUSE (mean %)	67.1	68.4	66.4	68.8

### Hospitalisation

**Figure 1:**
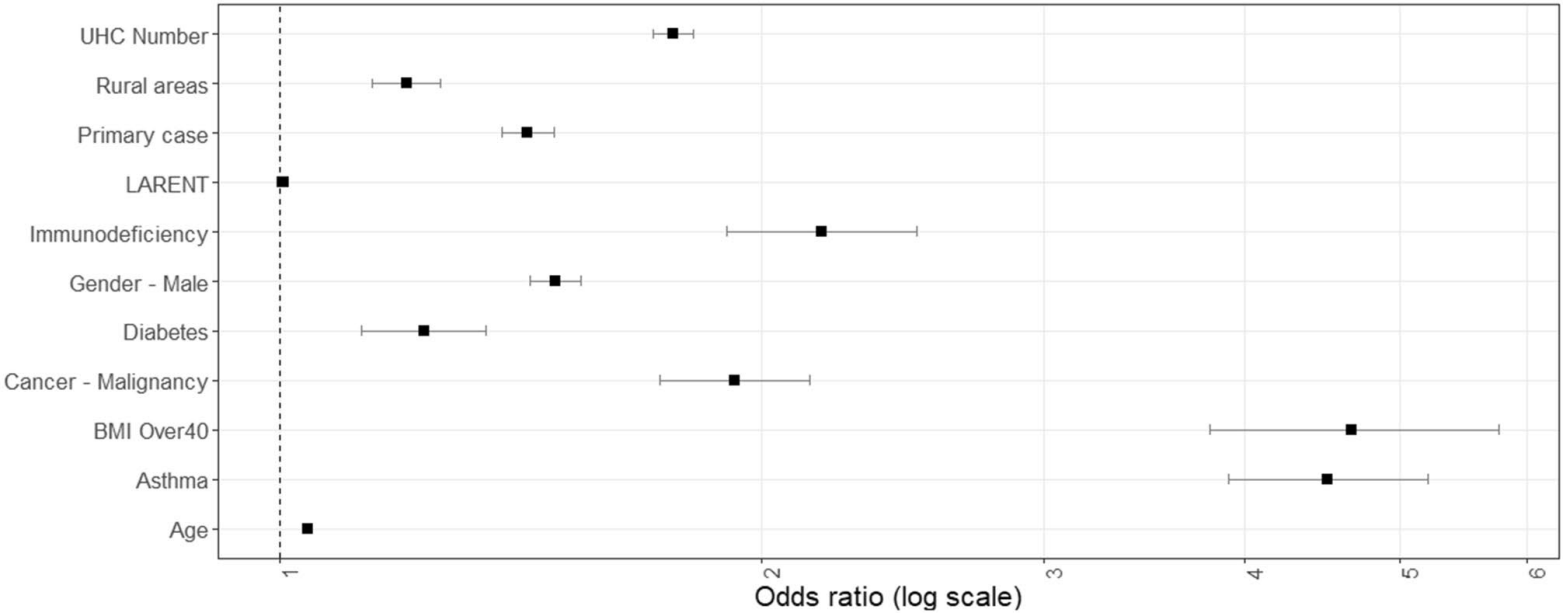
Forest plot of adjusted odds ratios for hospitalization from validated generalised linear models.

**Figure 2:**
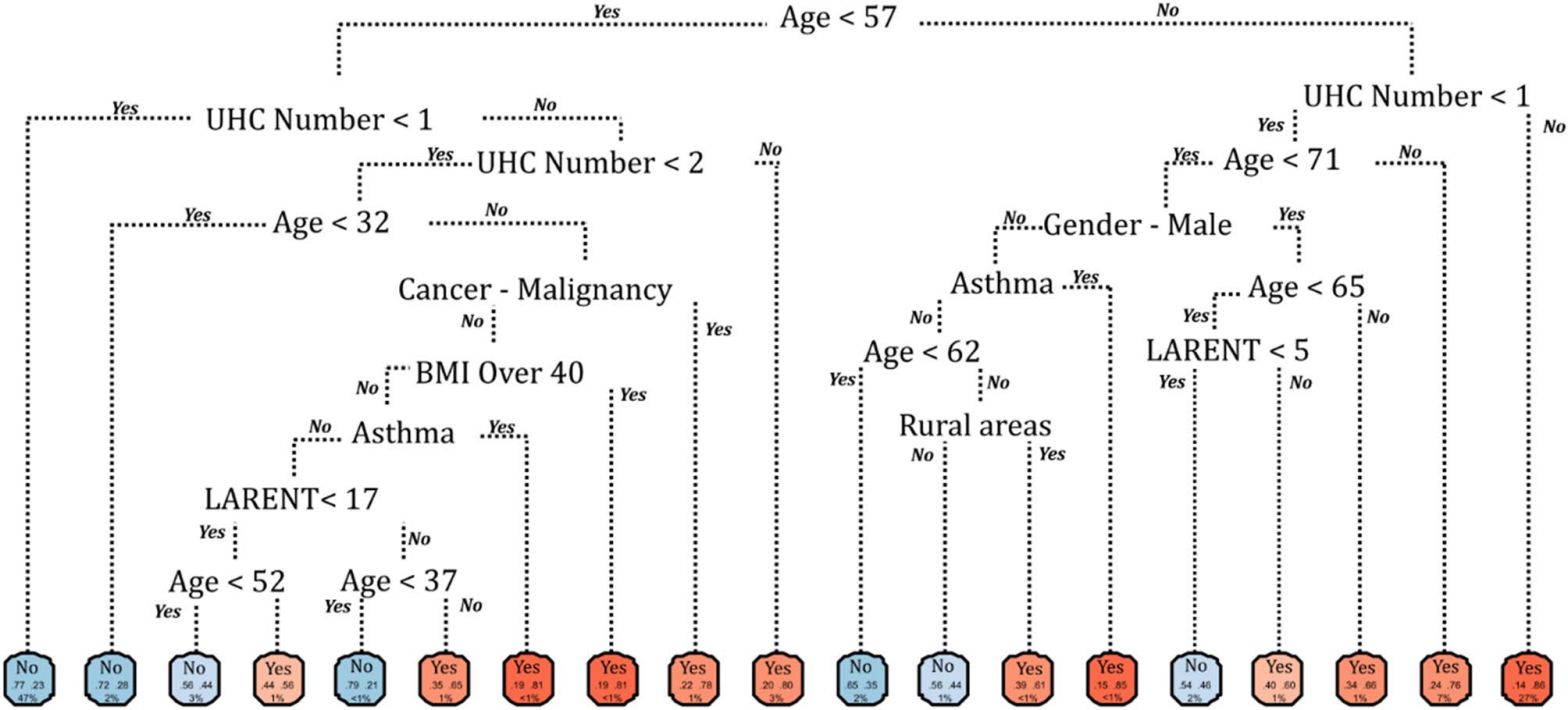
Recursive partitioning and regression tree (rtree) model for hospitalisation among symptomatic COVID-19 cases in the Republic of Ireland, February to November 2020.

All validated generalised linear models for severe COVID-19 outcomes are presented in Table 3. Almost 8% (n = 3781) of symptomatic COVID-19 infections during the study period resulted in hospitalisation; the validated GLM comprised 11 predictors, including age, gender, five individual comorbidities, calculated comorbidity number, primary case classification, and two geographically-specific variables (rurality, percentage of local authority housing) (Fig. 1; Table 3). The validated (tested) model (classification threshold 0.5) returned a bootstrapped (i.e., corrected) AUC of 0.816 (95% CI 0.809, 0.822), model predictive sensitivity (i.e., true positive) of 79.5%, Nagelkerke R^2^ of 0.411, and a Brier score of 0.166. The validated “rtree” model for hospitalisation among symptomatic COVID-19 cases is presented in Fig. 2; the model returned a predictive accuracy of 75.1% on the outcome (hospitalised) class.

**Table 3:** Generalised linear models predicting hospitalization, intensive care unit admission and mortality among symptomatic COVID-19 patients in the Republic of Ireland, February 29th to November 30th 2020 (N = 47,265).

	Hospitalization		ICU		Deaths	
	Coefficient (SE)	aOR (95% CI)	Coefficient (SE)	aOR (95% CI)	Coefficient (SE)	aOR (95% CI)
Age	0.042 (0.0005)	1.043 (1.042–1.044)	0.036 (0.001)	1.037 (1.036–1.038)	0.119 (0.001)	1.126 (1.124–1.129)
Asthma	1.505 (0.073)	4.504 (3.906–5.210)	2.011 (0.064)	7.467 (6.592–8.841)	0.516 (0.107)	1.676 (1.362–2.071)
Severe obesity (BMI ≥ 40)	1.540 (0.105)	4.663 (3.808–5.760)	2.977 (0.088)	19.630 (16.566–23.43)	2.380 (0.113)	10.802 (8.672–13.514)
Cancer/malignancy	0.654 (0.055)	1.922 (1.727–2.144)	–	–	1.113 (0.060)	3.044 (2.710–3.424)
Chronic neurological disease		–	− 1.501 (0.055)	0.223 (0.200–0.249)	–	–
Diabetes	0.208 (0.046)	1.231 (1.125–1.347)	0.256 (0.041)	1.191 (1.192–1.399)	–	–
Gender–Male	0.397 (0.019)	1.487 (1.434 − 1.542)	1.015 (0.021)	2.759 (2.647 ( 2.877)	0.354 (0.030)	1.425 (1.345–1.510)
Healthcare worker	–	–	− 0.241 (0.029)	0.786 (0.742–0.832)	− 1.901 (0.093)	0.149 (0.124–0.510)
Immunodeficiency	0.778 (0.069)	2.178 (1.903–2.497)	1.593 (0.061)	4.919 (4.372–5.546)	0.775 (0.095)	2.171 (1.803–2.621)
Local Authority housing (%)	0.006 (0.001)	1.006 (1.005–1.007)	–	–	–	–
Primary case	0.357 (0.019)	1.429 (1.376–1.485)	–	–	–	–
Rural Resident	0.183 (0.025)	1.200 (1.143–1.261)	–	–	–	–
UHC^1^ Number	0.566 (0.015)	1.761 (1.712–1.813)	0.904 (0.014)	2.469 (2.401–2.540)	0.771 (0.015)	2.162 (2.098–2.228)
Urban resident	–	–	0.474 (0.024)	1.533 (1.606–1.682)	–	-

^1^Underlying health condition.

### Admission to ICU

**Figure 3:**
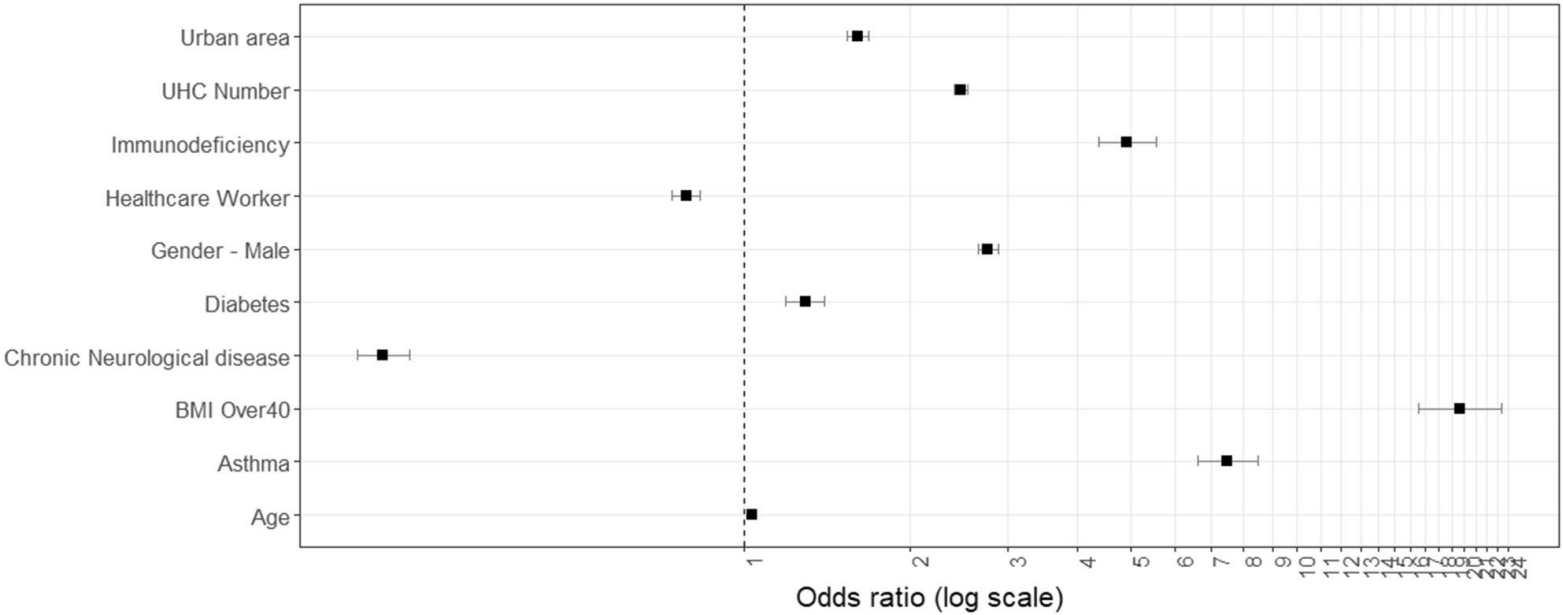
Forest plot of adjusted odds ratios for ICU admission from validated generalised linear models.

**Figure 4:**
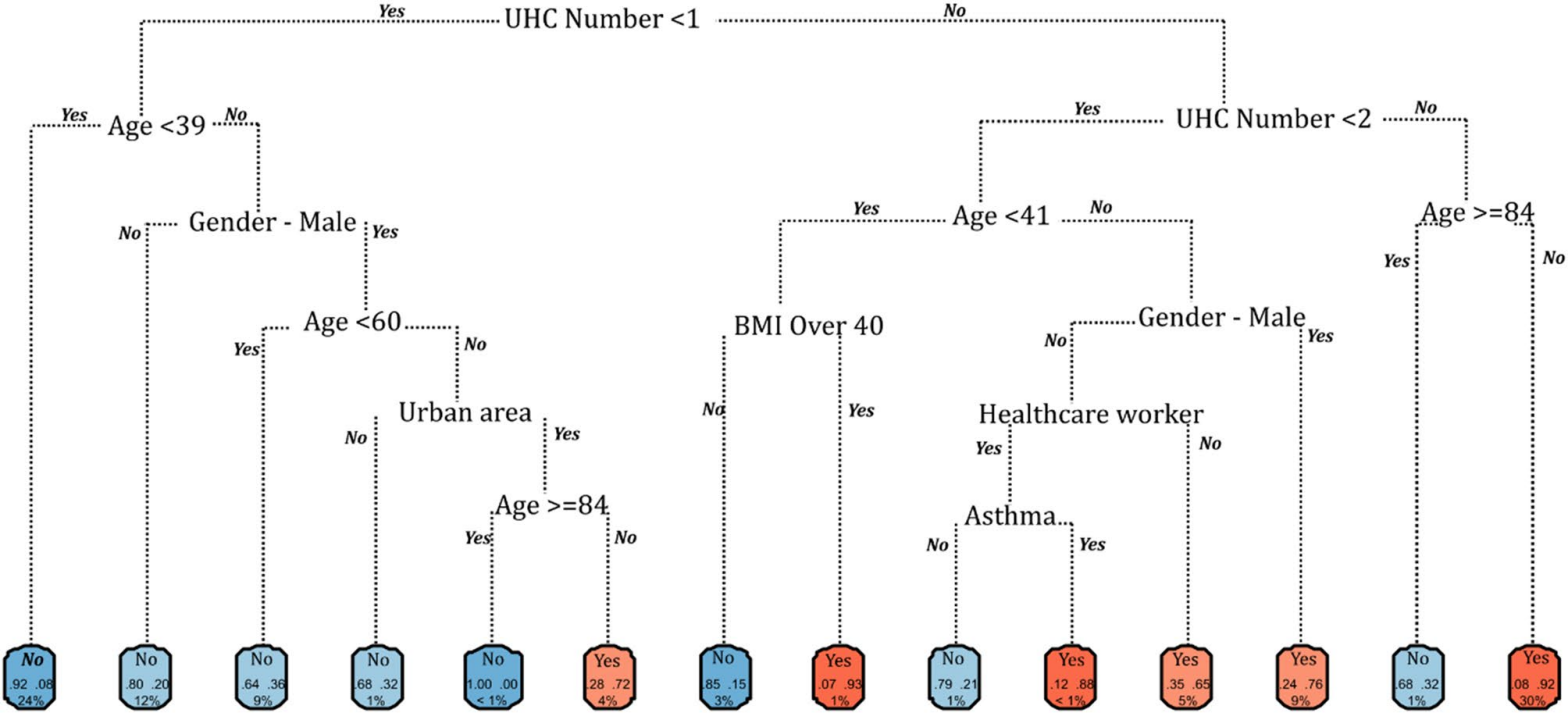
Recursive partitioning and regression tree (rtree) model for ICU admission among symptomatic COVID-19 cases in the Republic of Ireland, February to November 2020.

Approximately 1.3% (n = 615) of symptomatic COVID-19 infections from February 29th to November 30th resulted in admission to an ICU; the training GLM comprised 10 predictors, two of which were “protective” (healthcare worker, presence of a chronic neurological condition), including age, gender, five individual comorbidities, calculated comorbidity number, occupational classification, and one geographically-specific variable (urban resident) (Fig. 3; Table 3). The validated model returned a bootstrapped AUC of 0.885 (95% CI 0.88 0.89), model predictive sensitivity (i.e., true positive) of 85.2%, a Nagelkerke R^2^ of 0.575, and a Brier score of 0.128. The validated “rtree” model for hospitalisation among symptomatic COVID-19 cases is presented in Fig. 3; the model achieved a predictive accuracy of 83.1% on the outcome (ICU admission) class (Fig. 4).

### Mortality

**Figure 5:**
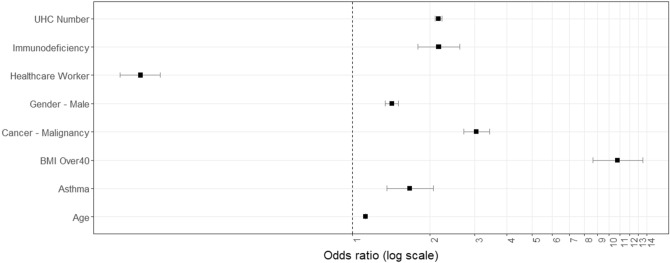
Forest plot of adjusted odds ratios for mortality from validated generalised linear models.

**Figure 6:**
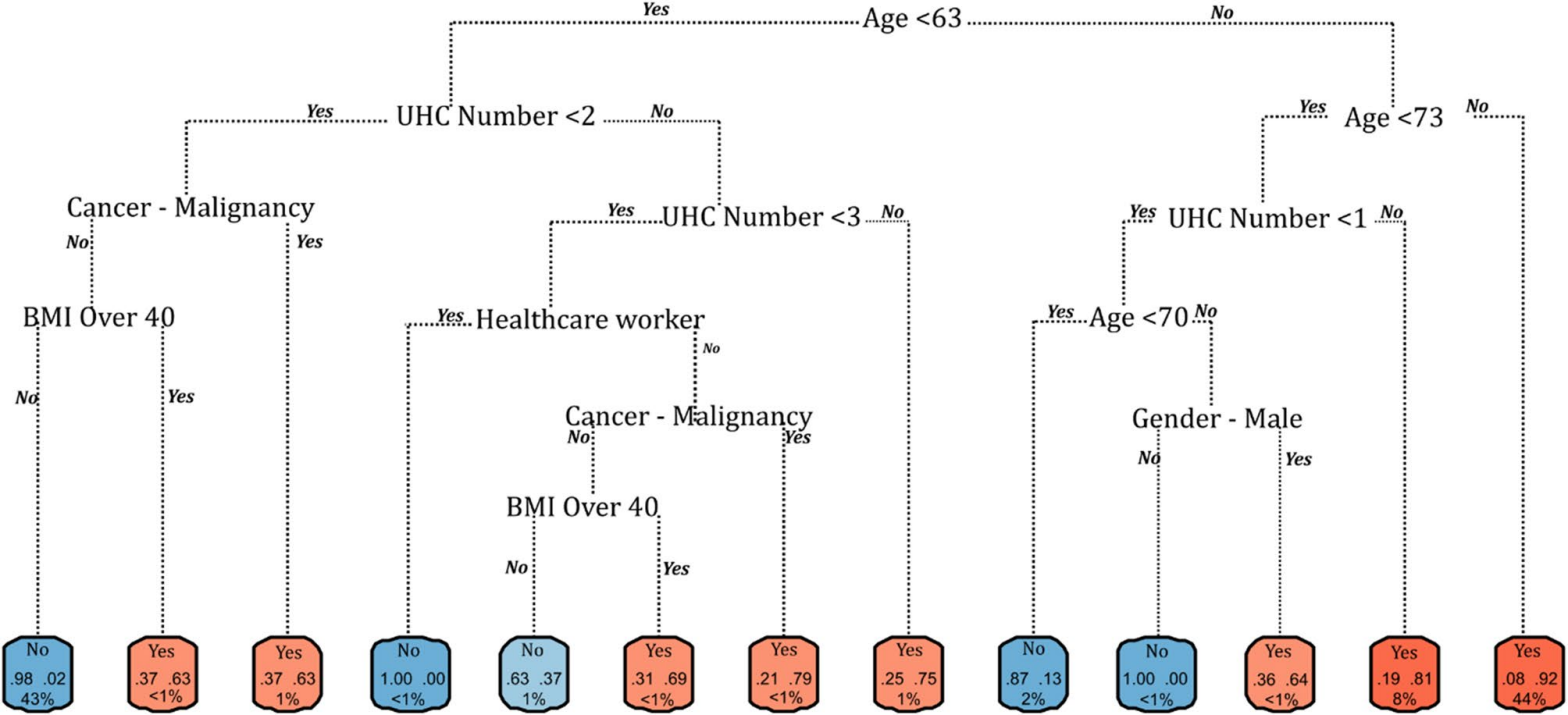
Recursive partitioning and regression tree (rtree) model for mortality among symptomatic COVID-19 cases in the Republic of Ireland, February to November 2020.

Just under 3% (n = 1326) of symptomatic COVID-19 infections occurring between February 29th and November 30th resulted in death; the validated GLM comprised 8 predictors, one of which was “protective” (healthcare worker), including age, gender, four individual comorbidities, calculated comorbidity number, occupational classification, and one geographically-specific variable (urban resident) (Fig. 5; Table 3). The validated model returned a bootstrapped AUC of 0.955 (95% CI 0.951 0.959), model predictive sensitivity (i.e., true positive) of 90.4%, a Nagelkerke R^2^ of 0.816, and a Brier score of 0.06. The validated “rtree” model for mortality among symptomatic COVID-19 cases is presented in Fig. 6; the model achieved a predictive accuracy of 96.7% on the outcome (mortality) class.

## Discussion

The complete Irish dataset of notified cases of COVID-19 throughout the first two waves of the pandemic was analysed to identify case- and geographically-specific attributes that may serve as predictors for hospitalization, ICU admission and mortality in patients with laboratory-confirmed, symptomatic COVID-19 infection. Results mirror findings from previous studies, with older age, male gender and increased comorbidity number consistently significant factors within all validated models for COVID-19 severity. Studies have shown that increasing type-2 cytokine production with age likely reduce control of viral replication, leading to prolonged incubation and inflammatory response, thus facilitating the progression of infection^16,17^. Likewise, while symptomatic COVID-19 prevalence was higher among females (53.4%), the burden of severe infection was markedly higher among male cases for all three modelled outcomes; men were approximately 1.5 (OR 1.487, 95% CI 1.434‒1.542)), 2.8 (OR 2.759, 95% CI 2.647‒2.877) and 1.4 (OR 1.425, 95% CI 1.345‒1.510) times more likely to be hospitalised, admitted to ICU, or die, than women within the study sample (OR 2.759, 95% CI 2.647‒2.877). A recent review of the sex- and gender-related differences associated with COVID-19 outcomes in Europe proposes numerous potential reasons for this relationship, including gender-specific lifestyle, health behaviours, psychological stress, and socioeconomic conditions, in addition to several sex-specific biological mechanisms modulating the course of disease, including hormone-regulated gene expression, innate and adaptive immune responses, and immune-aging^18^. For example, numerous studies have shown that females are generally less susceptible to viral infections and mount higher innate immune responses (more rapid viral recognition and type I interferon production) than their male counterparts, leading to faster viral clearance^19,20^. Accordingly, there is a strong evidence base to suggest that upon infection with SARS-CoV-2, females may be better equipped to initially respond, and attenuate viral invasion and pathogenicity compared to males. Additionally, a recent study in the UK has noted significantly higher rates of “behavioural resistance” to protection actions (i.e., non-pharmaceutical interventions) among men, noting that 80% of those fined for breaking lockdown measures were male^21^, potentially resulting in higher levels of viral exposure, transmission and loading among males, in concurrence with the aforementioned biological disparities. Accordingly, gendered or sex-specific therapies and/or non-pharmaceutical interventions may be an important area for future research.

COVID-19-related hospitalisation presented as the most analytically complex severe outcome, with numerous comorbidities and socioeconomic factors associated with admission as a hospital inpatient. While the models for predicting hospitalisation demonstrated a good fit (AUROC 0.816, 95% CI 0.809‒0.822), the authors suggest that the lower predictive capacity of the presented hospitalisation model is reflective of the complexity of disease manifestation, particularly within the community, which is mediated by several socio-behavioural, clinical and biological factors. This may be particularly pronounced with respect to non-clinical and non-biological factors such as individual behaviours, self-efficacy and knowledge, which may lead to increased exposures and are particularly difficult to accurately quantify via routine epidemiological surveillance.

Asthma was associated with an increased likelihood of hospitalization. Recent research has been divided regarding the influence of asthma on COVID-19 hospitalisation, with some authors suggesting that those with asthma are over-represented among adult hospital admissions as SARS-CoV-2 may initiate an exacerbation in asthma symptoms, which has been reported among other respiratory viruses^20,21^. Likewise, the most common presenting symptoms of COVID-19 — dry cough and shortness of breath — are also common in acute exacerbation of asthma^20^. Conversely, several international studies have reported that asthma is not a significant risk factor for hospitalisation with COVID-19^22,23^, with some suggesting that it may be a protective factor, via increased numbers of eosinophils in the airways of asthmatic patients, or through potential antiviral and immunomodulatory activities of inhaled asthma medications, and particularly steroids^24^. Results from the current study may reflect the high prevalence of asthma in the ROI, which has the fourth highest global prevalence of the disease and was consistently among the top 20 diagnoses for admission to hospital prior to the pandemic^25^.

From a socio-geographic/economic perspective, patients living in categorically rural areas and in regions characterised by higher (> 17%, Fig. 2) rates of local authority (i.e., publicly-supported) housing were also at increased risk of hospitalisation, potentially reflecting a geographical and/or geo-social gradient associated with disease severity in Ireland. A recent investigation of the socioeconomic association of COVID-19 hospitalisation among 418,794 participants of the UK Biobank reports a striking gradient in COVID − 19 hospitalization rates according to the Townsend Deprivation Index − a composite measure of socioeconomic deprivation − and household income^26^. Likewise, individual socioeconomic status has been associated with the severity of COVID-19 among hospitalised patients under the age of 70 years in Greater Paris, with housing conditions as they relate to the capacity to socially distance and increased co-resident infections, specifically mentioned as probable drivers^27^. Within the current study sample, local-authority housing (%) and the prevalence of both primary (R_sp_ = 0.375, *p* < 0.001) and college/university education (R_sp_ =  − 0.449, *p* < 0.001) were significantly correlated, with lower levels of education a globally recognised source of health inequalities^28^.

Predictive capacity increased for both ICU admissions and mortality, with models for ICU admission (AUROC 0.885, 95% CI 0.88–0.89) and mortality (AUC 0.955, 95% CI 0.95–0.96) assessed as being very good and excellent, respectively. Commonalities were observed across risk factors identified for both outcomes. Specifically, severe obesity, indicated by a body mass index (BMI) ≥ 40, was a significant marker for both ICU admission (OR 19.6) and death (OR 10.8). The identified risks associated with severe obesity align with pathophysiological mechanisms contributing to respiratory distress; in particular, a BMI ≥ 40 (associated with increased respiratory rate) is recognized as a contributor to multiple respiratory infections including pneumonia^29^ and has been identified as a primary risk factor for poor COVID-19 prognoses^30,31^. Severe obesity was a particularly significant predictor among COVID-19 patients aged < 41 years (Fig. 4) and < 63 years (Fig. 6) for ICU admission and death, respectively (i.e., significantly below median ages for both outcomes). Similarly, the presence of malignant cancer and immunodeficiency resulting from cancer treatment impair the ability to mount an effective response to clear viral infection and are associated with increased susceptibility to acute clinical deterioration and increased mortality due to increased viral pathogenicity^32,33^, with < 63 years again identified as a significant “splitter” for COVID-related mortality (Fig. 6) demonstrating a lack of interaction between this health condition and older age.

While residence in categorically rural areas was associated with a higher likelihood of hospitalisation, the opposite was true for admission to ICU, whereby urban dwellers were approximately 1.5 times more likely to require critical care (OR 1.533, 95% CI 1.606‒1.682), and particularly among those aged > 60 years (Fig. 4). Urban living may be indicative of multiple individual or interacting factors including higher levels of deprivation^34^, higher viral exposures (i.e., close contacts) due to increased household and/or local population density^26^ or compounded respiratory illnesses due to lower air quality in urban areas^35^. For example, within the current study sample, while a slightly higher proportion of symptomatic cases with asthma were reported in rural areas (~ 1.5% versus ~ 1% of all symptomatic cases), the likelihood of ICU admission among urban asthma sufferers was significantly higher (OR 15.55; CI 11.28–21.14) than their counterparts in rural (OR 13.22; CI 6.49‒25.10) or commuter areas (OR 11.01; CI 3.73‒26.99), potentially highlighting a significant interaction between urban pollutant exposures (e.g., particulate matter (PM) 2.5/10) and COVID-19 severity^36^.

The apparent “protective” effect of occupational status as a healthcare worker and both ICU admission and death may indicate clinical heterogeneity between these and other cases, arising from diagnostic bias, as healthcare workers are likely to be associated with a high index of suspicion for the disease, and as such, have and continue to undergo serial testing in Ireland. The threshold of clinical criteria for COVID-19 diagnosis in healthcare workers, and the temporal lag between viral exposure, positive diagnosis and subsequent treatment, is likely significantly lower than among the general population, due to these testing protocols^37^, resulting in improved outcomes and the apparent “protective” effect identified^38^. Likewise, the protective effect of chronic neurological disease with respect to ICU admission is thought to reflect clinical processes, specifically the clinical judgement that, regarding persons with advanced dementia, mechanical ventilation may prolong patient suffering without a clear survival benefit^39^. Other comorbidities have been associated with poor prognosis from ICU admission, and thus reduce odds of admission through clinical decision-making. Physical factors indicating a limited functional capacity are predictive of high mortality in ICU, suggesting that frailty has a significant impact on intensive care outcome; hence, the finding that age was associated with the lowest odds of ICU admission (compared with hospitalisation and mortality) may be unsurprising^8^. Similarly, the finding that 599 (45.2%) of those who died had not been hospitalised is unsurprising in the context of the mean age of this subgroup (84 years), as patients of such advanced age may have been considered too frail to benefit from hospital (and particularly critical) care.

While the presented study permits delineation of severe health outcomes based on clinical and socioeconomic attributes, there are some limitations the authors feel should be highlighted. Based on presented findings, it is likely that some case attributes may be indicative of differential healthcare access and thus not entirely elucidated by the pathophysiological mechanisms driving progression of the disease to increasing clinical severity and death. For example, rurality as a predictor of hospitalization, in conjunction with urban residence as a predictor for ICU admission, may reflect a lower threshold for rural residents to present to healthcare locations, and subsequently to be admitted for observation, to counter the risk of deterioration in the more remote home environment. Likewise, the choice of hospitalisation as a marker for COVID-19 severity also comprises some spatio-temporal limitations; hospitalisation itself may be affected by many factors including health-seeking behaviours, availability of care and healthcare policies or thresholds (e.g., more or less severe cases may be admitted to hospital and/or admission may be age-specific), which may be spatially unique and/or temporally fluid based on the capacity of a national or regional healthcare system to absorb cases (e.g., localised outbreaks). As such, the authors advise caution be exercised when comparing the current study findings with previous or future studies of a similar nature.

## Conclusion

The identified nationally-specific risks associated with demographic, underlying health (comorbidities), geographic location and socioeconomic profile, and the specific importance, attribute “splitters” and variable interactions represent a robust evidence base for development of increasingly targeted public-health recommendations, interventions and therapeutic approaches for high-risk groups, e.g., minimization of social contact among those with elevated BMI, urban asthma or immunodeficiency caused by cancer treatments, and thorough respiratory etiquette and hand hygiene among household contacts in specific settings and/or geographic regions. Moreover, communication of the scientific basis for ongoing and future interventions, and particularly geographically- or socioeconomically bespoke interventions may be used to combat pandemic fatigue and increase overall transparency and awareness of ongoing public-health events. Furthermore, the presented models offer a metric by which tailored vaccination schedules may be devised with prioritization by age, sex, co-morbidity status and region. Lastly, results presented offer valuable information for effective patient triage; identifying those at increased risk of disease progression and death based on a suite of factors and not solely on clinical presentations of the disease.
